# The influence of completing a health-related questionnaire on primary care consultation behaviour

**DOI:** 10.1186/1472-6963-6-101

**Published:** 2006-08-16

**Authors:** Amanda Jeffery, Clare Jinks, Kelvin Jordan

**Affiliations:** 1 Primary Care Musculoskeletal Research Centre, Keele University, Keele ST5 5BG, UK

## Abstract

**Background:**

Surveys of the population are commonly used to obtain information on health status. Increasingly, researchers are linking self-reported health status information to primary care consultation data. However, it is not known how participating in a health-related survey affects consultation behaviour. The objective of this study was to assess whether completion of a health-related questionnaire changes primary care consultation behaviour.

**Methods:**

Participants were 3402 adults aged 50 and over from the general population in North Staffordshire, UK, who completed a health-related postal survey received in April 2003. The survey was predominantly about occurrence and severity of knee pain in the last year. Primary care attendance for the three months following response was compared to three control periods: i) the three months prior to the survey, ii) the same time period in the previous year and iii) the same time period in the following year. Comparisons were made on consultations for any problem, consultations for musculoskeletal disorders and consultations for knee problems.

**Results:**

The percentage of subjects consulting for any condition was marginally higher for the three months directly after receipt of the questionnaire but the difference was only statistically significant in comparison to the three months before the survey (64% v. 62%, *p *= 0.05). There was little difference in consultation prevalence for musculoskeletal problems immediately after the survey compared to the three control periods. There was an increase of 37% in knee disorder consultations for the three months after the survey compared to the three months directly before the survey (*p *= 0.02). However, consultation prevalence for knee problems was identical for the three months after the survey to the same time periods in the years prior to and following the survey (both *p *= 0.94).

**Conclusion:**

The results from this study suggests that questionnaires related to physical health do not affect the standard consulting behaviour of patients, even for the symptom under investigation. This should reassure researchers who wish to link self-reported health status and medical care utilisation and clinicians whose patients are involved in such research.

## Background

Population health surveys are a common method of obtaining information on the extent and severity of self-reported health problems. Further information on the occurrence and treatment of symptoms and disease can be obtained from primary care consultation records. The increased recording of general practice data electronically means that this information is becoming more readily available and hence an increasingly important resource for researchers. This also means there is increased opportunity for linking self-reported health status with recorded consultation data for responders who consent to access to records. For example, we have previously combined these data sources in order to determine predictors of general practice consultation for knee pain [1].

Whilst the factors associated with consenting to medical record review have been examined [2-6], there is little information on the impact that the receipt of a health-related questionnaire has on health care utilisation, in particular for the symptom under investigation. One study suggested an increased incidence of disease following reporting back of clinical measurements taken as part of a survey [7]. However, it is important for researchers investigating the link between self-reported health status and medical care utilisation to know whether the completion of health-related questionnaires affects the usual consulting behaviour of participants. Clinicians may also be uncertain of the impact that such research within their practice may have on their workload.

The objective of this study was to assess whether the completion of a health-related questionnaire, within a study of knee pain, changes primary care consultation behaviour by comparing responders' consultation rates before and after a postal survey.

## Methods

Participants in this study had been part of a knee pain survey (KNEST) involving all adults aged 50 and over registered at three general practices in North Staffordshire, U.K [8,9]. They had completed a follow up questionnaire sent in April 2003 (three years after a baseline survey), consented to viewing of their medical records and were still registered with the practices at the end of July 2004. The study was approved by the North Staffordshire Local Research Ethics Committee.

The three practices involved are part of the North Staffordshire and Central Cheshire General Practice Research Network. All practices undergo regular training, assessment and feedback in the quality of their computerised morbidity coding [10]. Morbidities are entered onto the computer in these practices using the Read Code classification, a hierarchy of morbidity, symptom and process codes commonly used in the U.K. [11]. Further information on a consultation can be input into a "free text" field.

A consultation was defined as a visit to the surgery, a home visit or contact by telephone. Comparisons were made on all consultations, musculoskeletal consultations and knee-related consultations. When defining all consultations, only one problem was counted if there were multiple problems coded on one day. Repeat consultations for the same problem on different days were included. Musculoskeletal consultations were defined as a consultation coded under Chapter N (Musculoskeletal and Connective Tissue disorders) of the Read Code system. Knee consultations were defined using Read Codes classified independently by two GPs and by a search for the word knee in the consultation text by two researchers independently. Disagreements were resolved by consensus.

The percentage of responders consulting at least once for the three month period following the survey (May–July 2003) was compared to the same three month (May–July) periods in 2002 and 2004, and for the three month period prior to the survey (Jan–Mar 2003). A three month period was chosen for analysis to allow time for the mailing process to be completed (for example, to include the mailing of two week and four week reminders to non-responders) and to allow sufficient time for any effect of the questionnaire to result in an actual consultation. Changes in consultation status for participants were assessed using McNemar tests. The total number of consultations made by the participants in each time period were calculated as a rate per 10000 people.

## Results

At follow-up, 5784 subjects were sent a questionnaire. 4317 (adjusted response 75%) responded of whom 3543 (82%) had consented to medical record review. 3402 participants were still registered at the practices at the end of July 2004, 83 had died and 58 were no longer registered at the practices. The 3402 participants form the study population for this analysis. The mean age of the subjects was 67.3 (SD 9.10) at the time of follow-up and 1909 (56%) were female. Compared to the remainder of the subjects who were sent a questionnaire, the 3402 participants were similar in terms of gender (*p *= 0.44), were slightly younger (mean difference 1.2 yrs; 95% CI 0.7, 1.7), and had higher prevalences of self-reported knee pain in the last year at baseline (48% v 45%, *p *= 0.006) and of any pain in the last month at baseline (69% v 62%, *p *< 0.001). They also had a slightly higher prevalence of self-reported knee pain in the last year at follow-up (*p *= 0.05), and of any pain in the last month at follow-up (*p *= 0.002), than those who responded at follow-up but did not consent to viewing of medical records.

**Figure 1:**
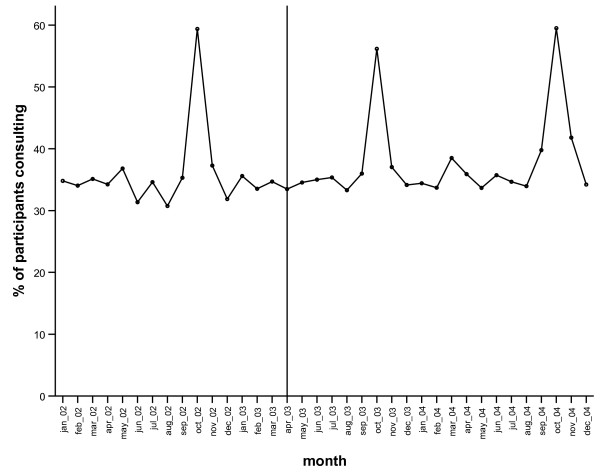
The percentage of patients consulting for any problem by month.

Figure 1 shows the percentage of subjects consulting at least once for any condition in each month between January 2002 and December 2004. These monthly consultation rates showed little change after the survey in April 2003 to the months immediately before the survey took place. Consultation rates for October of each year are much higher due to receipt of influenza vaccinations.

**Figure 2:**
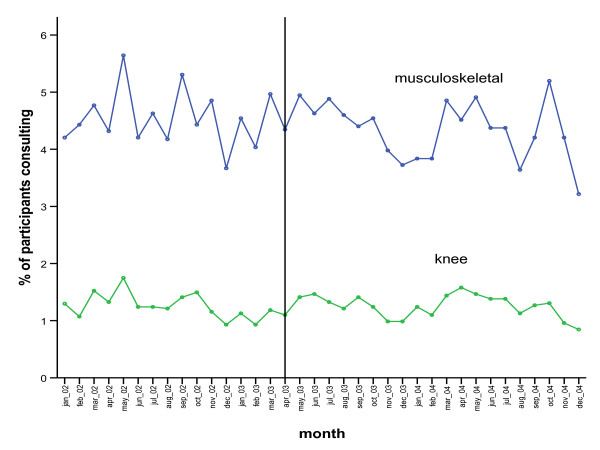
The percentage of patients consulting for i) a musculoskeletal disorder and ii) a knee problem by month.

The percentage of subjects who consulted at least once for any problem in the three months immediately after the survey (64%) was slightly higher than in the three months prior to the survey (62%) and in the corresponding three months in the years prior to and after the survey (both 62%) (table 1). These small differences were also apparent when analysing only musculoskeletal consultations. However, only the comparison on all consultations with the three months immediately before the survey was close to statistical significance (difference in proportions 2.0%; 95% CI 0.0%, 3.9%; McNemar test, *p *= 0.05). The monthly musculoskeletal consultation prevalence rates fluctuated mainly between 4–5% and show little change after the survey (figure 2).

The number of participants consulting for the knee rose by more than a third (37%, McNemar test, *p *= 0.02) in the three months after the survey compared to the three months before the survey but was virtually identical to the number consulting during the same time period (May–July) in the years before and after the survey. The percentage of subjects consulting for the knee appear to follow a cyclical pattern with lower consultation figures for the winter months in each year (figure 2).

Total number of consultations in the three months after the survey was similar or lower than in the other three time periods (table 1).

## Discussion

In this large study of responders to a health status questionnaire there was a slight increase in the percentage of participants consulting primary care following the survey. There was a sharper increase in knee consultations, the condition under study. However, this appeared to be due to a cyclical pattern rather than the effects of participating in the survey as the number of people consulting for the knee immediately following the survey was almost identical to that in the same time period in the years before and after the survey.

There has been little study into the effect of a health-related questionnaire on consultation rates. One study showed an increase in health care recorded incidence of diabetes, hypertension and elevated cholesterol following reporting to participants of blood pressure and laboratory results collected as part a health survey [7]. That study suggested surveys about health might directly influence subsequent consultation. Our study suggests this is not true for knee pain. The difference may be explained in that our study involved a postal health-related questionnaire, whereas the previous study involved a face-to-face survey with feedback of clinical and laboratory results. Results from these procedures may have instilled more concern in the respondent and hence led to consultation.

Knee pain, whilst common in the elderly, does not often initiate consultation in the elderly even in those with severe pain [9]. Therefore, it could be hypothesised that the receipt of a questionnaire specifically targeted at knee pain may highlight the problem to the respondent, and lead to more consultation. This does not appear to be the case.

This study explored the effect of a questionnaire specifically addressing the physical health of respondents. It is possible that a questionnaire focussing on mental health problems may lead to increased consultations for mental disorders. This is an area for future research.

This was a large study examining changes in consultation patterns before and after a survey of 3402 responders and, hence, able to detect small changes in consultation prevalence. This is evidenced by the statistically significant result for consultation for knee problems in the three months after the survey compared to the three months before the survey. This pattern of increased consultation compared to the three months directly before the survey was evident in the same three months within the non-survey years. The survey had a high response rate and a high consent to medical record review. Although the practices were spread across a range of socio-economic areas, they are all in one region of the UK (North Staffordshire) which may effect generalisability. We have focussed on completion, as opposed to receipt, of health-status questionnaires. It may be that non responders and subjects who responded but did not consent to viewing of their medical records increased their use of health care services following receipt of the questionnaire. This is unlikely to have a large impact on health service use. In any case, comparison of those in our study to non-responders and non-consenters to medical record review suggest that the subjects in our study had slightly worse physical health and, therefore, may be more inclined to use health services. Previous studies have shown consenters to medical record review have slightly higher consultation rates than the remainder of their practice populations, non responders, and non consenters during the year after the study [2,4] and that participants who consent to medical record review are more likely to have the symptom under investigation and poorer health in general [3-5]. Whilst there may be some concern over whether consenters to medical record review are representative of the target population, they do not appear to change their consultation behaviour following completion of a health-related questionnaire.

## Conclusion

This study has shown that completion of a questionnaire related to physical health does not influence the consulting behaviour of participants in research studies. This should give researchers linking consultation data to survey data more confidence that consultation behaviour has not been affected by the survey. It should also reassure clinicians that becoming involved in such research is not going to have adverse effects on their workload. The effect of a survey on consultation may now be usefully considered for studies focussing on other morbidities.

## Competing interests

The author(s) declare that they have no competing interests.

## Authors' contributions

AJ carried out the analysis and drafted the manuscript. CJ conceived the design of the study, coordinated the study and participated in revision of the manuscript and interpretation of data. KJ also carried out analysis and participated in drafting the manuscript and in interpreting the data. All authors read and approved the final manuscript.

## Pre-publication history

The pre-publication history for this paper can be accessed here:



## Figures and Tables

**Table 1:** Comparison of general practice consultation prevalence rates before and after a postal health-related survey of people aged 50 and over living in the community

	May–July 2003^a^	Jan–Mar 2003^b^	May–July 2002	May–July 2004
*n *(%) of patients consulting				
all problems	2162 (64)	2095 (62) *p *= 0.05	2116 (62) *p *= 0.18	2112 (62) *p *= 0.14
musculoskeletal problems	397 (12)	369 (11) *p *= 0.25	381 (11) *p *= 0.54	381 (11) *p *= 0.54
knee problems	123 (4)	90 (3) *p *= 0.02	121 (4) *p *= 0.94	125 (4) *p *= 0.94
				
No. of consultations (all) per 10000 people	14377	14383	14547	15726

^a ^three months immediately after survey

^b ^three months immediately before survey

*p*-values are based on McNemar tests and compared consultation in each three month period to the three months after the survey (May–July 2003)
